# A structural investigation of NRZ mediated apoptosis regulation in zebrafish

**DOI:** 10.1038/s41419-018-0992-0

**Published:** 2018-09-20

**Authors:** Chathura D. Suraweera, Sofia Caria, Michael Järvå, Mark G. Hinds, Marc Kvansakul

**Affiliations:** 1 Department of Biochemistry and Genetics, La Trobe Institute for Molecular Science, La Trobe University, Melbourne, Victoria 3086 Australia; 2 Bio21 Molecular Science and Biotechnology Institute, The University of Melbourne, Parkville, Australia

## Abstract

Bcl-2 family proteins play a crucial role in regulating apoptosis, a process critical for development, eliminating damaged or infected cells, host-pathogen interactions and in disease. Dysregulation of Bcl-2 proteins elicits an expansive cell survival mechanism promoting cell migration, invasion and metastasis. Through a network of intra-family protein–protein interactions Bcl-2 family members regulate the release of cell death factors from mitochondria. NRZ is a novel zebrafish pro-survival Bcl-2 orthologue resident on mitochondria and the endoplasmic reticulum (ER). However, the mechanism of NRZ apoptosis inhibition has not yet been clarified. Here we examined the interactions of NRZ with pro-apoptotic members of the Bcl-2 family using a combination of isothermal calorimetry and mutational analysis of NRZ. We show that NRZ binds almost all zebrafish pro-apoptotic proteins and displays a broad range of affinities. Furthermore, we define the structural basis for apoptosis inhibition of NRZ by solving the crystal structure of both *apo*-NRZ and a *holo* form bound to a peptide spanning the binding motif of the pro-apoptotic zBad, a BH3-only protein orthologous to mammalian Bad. The crystal structure of NRZ revealed that it adopts the conserved Bcl-2 like fold observed for other cellular pro-survival Bcl-2 proteins and employs the canonical ligand binding groove to bind Bad BH3 peptide. NRZ engagement of Bad BH3 involves the canonical ionic interaction between NRZ R86 and Bad D104 and an additional ionic interaction between NRZ D79 and Bad R100, and substitution of either NRZ R86 or D79 to Ala reduces the binding to Bad BH3 tenfold or more. Our findings provide a detailed mechanistic understanding for NRZ mediated anti-apoptotic activity in zebrafish by revealing binding to both Bad and Noxa, suggesting that NRZ is likely to occupy a unique mechanistic role in zebrafish apoptosis regulation by acting as a highly promiscuous pro-apoptotic Bcl-2 binder.

## Introduction

Multicellular organisms have evolved a multitude of mechanisms to remove superfluous cells^1^. Pivotal among the mechanisms for cell removal is programmed cell death or apoptosis, a process that maintains tissue homeostasis, removes damaged, infected in response to pathogen invasion^2^, or otherwise unwanted cells, such as during embryonic development, where it plays a critical role in shaping body and tissue structures^3,4^. Members of the B-cell lymphoma-2 (Bcl-2) family of proteins are key players of cellular life and death decisions and regulate the intrinsic or mitochondrial associated cell death^3,4^. Consisting of ~20 proteins the Bcl-2 family is characterized by the presence of conserved sequence motifs referred to as Bcl-2 homology or BH motifs. Structurally, the Bcl-2 proteins are organized into two major sub-families, those that share the Bcl-2 fold (Bcl-2, Bcl-x_L_, Bcl-w, Mcl-1, A1, Bcl-B, Bax, Bak Bok) and a distantly related group, the BH3-only proteins that bear only a BH3-motif (Bim, Bad, Bmf, Bid, Bik, Hrk, Puma and Noxa) that with the exception of Bid are disordered^5^. Those with pro-survival activity in mammals comprise Bcl-2, Bcl-x_L_, Bcl-w, Mcl-1, A1 and Bcl-B. Their primary function is to antagonize the activation of caspases by directly interacting and inhibiting pro-apoptotic Bcl-2 proteins^4,6^ of the BH3-only group or Bax, Bak and Bok. It is ultimately the balance and specificity^7^ of interactions between pro-apoptotic and pro-survival Bcl-2 proteins that regulates apoptosis and determines cellular fate^8^.

The pro-apoptotic Bcl-2 proteins promote apoptosis via mitochondrial outer membrane permeabilization (MOMP)^4,5^. Critical to the execution of MOMP are Bax and Bak^9,10^ as they trigger formation of oligomeric pores that breach the mitochondrial outer membrane and release pro-apoptogenic molecules such as SMAC/DIABLO and cytochrome-*c*^11^ into the cytoplasm to activate the caspase cascade that proteolyses key cellular components and ultimately demolishes the cell^4^. In contrast to Bax and Bak, the BH3-only proteins induce apoptosis by utilizing their BH3 motif via two mechanisms: either indirectly by neutralizing pro-survival Bcl-2 by binding to a conserved ligand binding groove or directly by interacting with Bax and Bak via an alternative interaction site^5,12^. In healthy cells, BH3-only proteins act as sentinels of cellular well-being and are up-regulated in response to cellular insults including growth factor deprivation, exposure to cytotoxic drugs or viral infections, leading to the activation of cell death mechanisms^6^. Other less well described functions have also been attributed to the Bcl-2 family. For example, Bcl-2 family members may regulate or monitor intracellular calcium^13,14^ in the unfolded protein response (UPR) and trigger apoptosis through activation of the BH3-only proteins^15^ when the unfolded protein levels in the endoplasmic reticulum (ER) become excessive^16^. While elements of the intrinsic apoptotic pathway are highly conserved from sponges^17^ to mammals^6^ there are also significant differences between organisms^18^.

Many Bcl-2 family members are conserved between fish and mammals^19–21^, but some notable differences exist between the apoptotic machineries in mammals and fish. For instance, overexpression of the zebrafish (*Danio rerio*) pro-apoptotic Bcl-2 proteins zBmf1, zBik, zPuma and zNoxa triggered dose-dependent caspase activation and subsequent cell death, whereas overexpression of zBad and zBok did not lead to cell death compared to overexpression of human Bad^22^. Also, expression of zebrafish *bad* gene did not result in embryonic lethality^23^. Furthermore, zBad pro-apoptotic activity is regulated through phosphorylation of conserved serine residues^23,24^, in a manner similar to mouse Bad^25^. One significant difference from mammals is that zebrafish lack orthologues of the pro-survival Bcl-2 proteins Bcl-w and A1, as well as pro-apoptotic Bak and Hrk^20^, but contain a novel pro-survival protein called NRZ, that was initially identified as an orthologue of the avian pro-survival Bcl-2 protein NR-13^26–28^. Also present is a recently described pro-apoptotic Bcl-2 protein Bcl-wav^29^ that is without a mammalian orthologue. Thus, there are a number of significant differences between Bcl-2-regulated apoptosis in zebrafish and mammals.

NRZ is a 19 kDa protein expressed in the epiboly of the extra-maternal yolk syncytial layer (YSL) of zebrafish eggs^28^. In vivo, NRZ has been shown to be a potent inhibitor of apoptosis after serum withdrawal, and controls development during somatogenesis and gastrulation^28^. Mechanistically, NRZ was shown to inhibit apoptosis by antagonizing zBax-BH3^28^. *Nrz* knockout triggers intracellular Ca^2+^ increase in YSL, which results in the blockade of development prior to gastrulation^13^. Additionally, NRZ appears to arrest Ca^2+^ release from endoplasmic reticulum (ER) by direct interaction with the inositol triphosphate type 1 receptor, IP3R1, calcium channel^13^. Knockdown of NRZ is lethal in zebrafish embryos, and intriguingly, some of this activity appears to be independent of its role in apoptosis^28^. Loss of NRZ prevents embryonic development via upregulation of the transcription factor Snail-1, a cell adhesion regulator to arrest gastrulation at the shield stage^28,30^. Other activities ascribed to NRZ include inhibition of the apoptosis accelerating function of Bcl-wav, a pro-apoptotic Bcl-2 member found in teleost fish^29^.

Although there are significant differences between the apoptosis machinery of zebrafish and mammals there remains a high degree of conservation with many direct orthologues of mammalian apoptotic genes present in the genome of the zebrafish^19,20^. Coupled with the presence of many orthologous mammalian genes are the advantages of zebrafish as a model organism, such as their rapid development, embryo transparency and genetic accessibility^20^. Analysis of zebrafish genetics is providing a better understanding of the fundamental interactions governing apoptosis and is of significant interest in deciphering human disease, including cancer^20^ and host-pathogen interactions^31^. Here we report the first systematic biochemical analysis and high-resolution structure determination of a zebrafish pro-survival Bcl-2 protein, NRZ. Our findings suggest that NRZ is a unique pro-survival Bcl-2 protein with an unusual pro-apoptotic Bcl-2 binding profile unlike its counterparts in mammalian systems.

## Materials and methods

### Protein expression and purification

Synthetic cDNA encoding for codon-optimized NRZ (Uniprot Accession number Q8UWD5) lacking the 28 C-terminal residues (Bioneer, Melbourne, Australia) was cloned into the bacterial expression vector pCoofy4^32^ Recombinant NRZ was expressed in BL21-CodonPlus cells using the auto-induction method^33^ for 24 h at 25 °C with shaking. Bacterial cells were collected by centrifugation at 4000 rpm (JLA 9.1000 rotor, Avanti J-E Beckman Coulter, Mount Waverly, Australia) for 20 min and re-suspended in 50 ml lysis buffer A (50 mM Tris, pH 8.5, 300 mM NaCl and 2 mM BME (β-Mercaptoethanol) supplemented with lysozyme and DNaseI. The cells were lysed using sonication (programme 7, Model 705 Sonic Dismembrator, Fisher Scientific, Hampton, New Hampshire, US) and the resultant lysate was transferred into SS34 tubes for further centrifugation at 16,000 rpm (JA-25.50 rotor, Beckman Coulter Avanti J-E) for 30 min. The supernatant was loaded onto a HisTrap HP, 5 ml (GE Healthcare, Little Chalford, UK) equilibrated with buffer A. After sample application, the column was washed with 100 ml of buffer B (50 mM Tris, pH 8.5, 300 mM NaCl, 25 mM imidazole and 2 mM BME (β-Mercaptoethanol) and the target protein was eluted with buffer C (50 mM Tris, pH 8.5, 300 mM NaCl, 300 mM imidazole and 2 mM BME (β-Mercaptoethanol)) followed by HRV 3C protease cleavage while dialyzed overnight into buffer A at 4 °C. The cleaved protein was passed again through the column to remove the cleaved His-MBP tag, with the remaining protein being concentrated using a centrifugal concentrator with 10 kDa molecular weight cutoff (Amicon® Ultra 15) to a final volume of 4 ml. Concentrated NRZ was subjected to size-exclusion chromatography using a Superdex S75 16/600 column mounted on an ÄKTAXpress system (GE Healthcare) equilibrated in 25 mM HEPES, pH 7.5, 150 mM NaCl, 2 mM BME where it eluted as a single peak. The final sample purity was estimated to be higher than 95% based on SDS–PAGE analysis. Appropriate fractions were pooled and concentrated using a centrifugal concentrator with 10 kDa molecular weight cutoff (Amicon ® Ultra 15) to final concentration of 28 mg ml^−^^1^.

#### Expression and purification of NRZ mutants R86A and D79A

NRZ mutants D79A and R86A were codon-optimized and synthesized (Genscript) and subsequently cloned into the pGEX-6P-3 vector (Invitrogen). Expression and purification were performed using the same protocol as for wild-type NRZ.

#### Measurement of dissociation constants

Binding affinities were measured by isothermal titration calorimetry (ITC) employing a MicroCal iTC200 system (GE Healthcare) at 25 °C using wt-NRZ, and NRZ mutants D79A and R86A in 25 mM HEPES, pH 7.5, 150 mM NaCl, 2 mM BME at a final concentration of 30 μM as previously described^34^. BH3 motif peptide ligands were used at a concentration of 300 μM and titrated using 19 injections of 2.0 μl of ligand. All affinity measurements were performed in triplicate. Protein concentrations were measured using a Nanodrop UV spectrophotometer (Thermo Scientific, Scoresbury, Australia) at a wavelength of 280 nm. Peptide concentrations were calculated based on the dry peptide weight after synthesis. The zebrafish BH3-motif peptides used were commercially synthesized and were purified to a final purity of 95% (GenScript, Piscataway, New Jersey, US). zBim: ALPPEMVVARELRRIGDEFNRLYCEA (UniProt accession code B2KKY9, residues 117–142), zPuma: EEQAVERVAVQLRTIGDEMNAVFLQR (accession code Q0GKC9, residues 123–148), zBik: NMRVTQTIGRQLAQIGDEMDNKWRQE (accession code Q5RGV6, residues 30–55), zBax: ELCDPSHKRLAQCLQQIGDELDGNAQLQ (accession code Q9I9N4, residues 52–79), zBid: EARAAREMAAELIRIADLLEQSVLSQAA (accession code Q0GKC5, residues 85–112), zBad: ALWAAKKYGQQLRRMSDEFDKGQMKR (accession code A7MCM4, 88–113 residues), zBmf: AQSVETLIGQKLQLIGDQFYQEHIMH (accession code Q0GKC7, residues 89–114), zNoxa: EQTAVVECAQQLRNIGDLLNWKYKLL (accession code Q0GKC8, residues 5–30), zBok: PRGVLVDVSVVLLKLGDELECMRPYV (accession code Q7T381, residues 60–85), zBeclin: DGGTMENLSRRLKVTSNLFDIMSGQT (accession code F1RCP1, residues 102–127), zBcl-wav: LCPAPSRASAALRHAGDELLIRFPIF (accession code D2Y5Q2, residues 42–67).

#### Crystallization and data collection

Crystals of *apo* NRZ were obtained at a protein concentration of 28 mg/ml in 1.0 M magnesium sulphate hydrate, 0.1 M sodium acetate trihydrate pH 4.6. The crystals were cryo-protected in mother liquor supplemented with 30% glucose and flash cooled in liquid nitrogen. The *apo* NRZ crystals in this condition appeared as thick needles belonging to the P4_3_ space group of the tetragonal crystal system.

All diffraction data were collected on the MX2 beamline at the Australian Synchrotron using an Eiger detector (Dectris, Baden-Dättwil, Switzerland) with an oscillation range of 0.1° per frame using wavelength 0.9537 Å. The diffraction data were integrated using XDS^35^ and scaled using AIMLESS^36^. The crystals of *apo-*NRZ contained one molecule of NRZ in the asymmetric unit with a calculated solvent content of 47.0%. The structure of *apo*-NRZ was solved by molecular replacement using PHASER^37^ with previously solved structure of NRZ: Bad BH3 (PDB ID: 6FBX) as a search model. The final TFZ and LLG values were 15.4 and 541.6, respectively. The final *apo*-NRZ structure was built manually over multiple cycles using Coot^38^ and refined using PHENIX^39^ to a final *R*_work_/*R*_free_ of 0.194/0.222 with 96.3% of residues in Ramachandran favoured region of the plot and no outliers. All data collection and refinement statistics are summarized in Table 2.

Complexes of NRZ with zBad BH3 were prepared as previously described^40^. Briefly, NRZ: zBad BH3 complexes were reconstituted by adding zBad BH3 peptides at a 1:1.25 molar ratio to NRZ. The reconstituted complex was concentrated to 28 mg ml^−1^ using a 3 kDa molecular weight cutoff centrifugal concentrator (Millipore), flash-cooled and stored under liquid nitrogen. High-throughput sparse matrix screening was carried out using 96-well sitting-drop trays (Swissci, Neuheim, Switzerland) and the vapour-diffusion method at 20 °C. Crystals of NRZ: zBad BH3 were obtained at 28 mg ml^−1^ using the sitting-drop method at 20 °C in 0.2 M sodium fluoride, 0.1 M Bis-Tris propane, pH 6.5, 20% (W/V) PEG 3350. The crystals were flash-cooled at −173 °C in mother liquor supplemented with 30% (w/v) glucose. The NRZ: zBad BH3 complex formed single rod-shaped crystals belonging to space group P6_3_ in the hexagonal crystal system.

Diffraction data for NRZ: zBad BH3 complex were collected on the MX2 beamline at the Australian Synchrotron using a with an Eiger detector with an oscillation range of 0.1° per frame using wavelength 0.9537. Collected diffraction data were integrated using XDS^35^ and scaled using AIMLESS^36^. Molecular replacement was performed using PHASER^37^ with the structure of Mcl-1 (PDB ID: 5KU9)^41^ as a search model. NRZ: zBad BH3 crystals contain one molecule of NRZ and 1 molecule of zBad BH3 in the asymmetric unit, with a 43.7% solvent content and final TFZ and LLG values of 9.2 and 63.76, respectively. The final model of NRZ: zBad BH3 was built manually over several cycles using Coot^38^ and refined using PHENIX^39^ with a final R_work_/R_free_ of 0.187/0.206, with 98.7% of residues in Ramachandran favoured region of the plot and no outliers.

All images for NRZ *apo* and NRZ: zBad BH3 complex were generated using PyMOL molecular graphic system, version 1.8.6 (Schrödinger, LLC, New York, USA). All software was accessed through the SBGrid suite^42^.

### Sequence alignments

Sequence alignments were performed using MUSCLE (https://www.ebi.ac.uk/Tools/msa/muscle/) with the default settings.

## Results

**Fig. 1:**
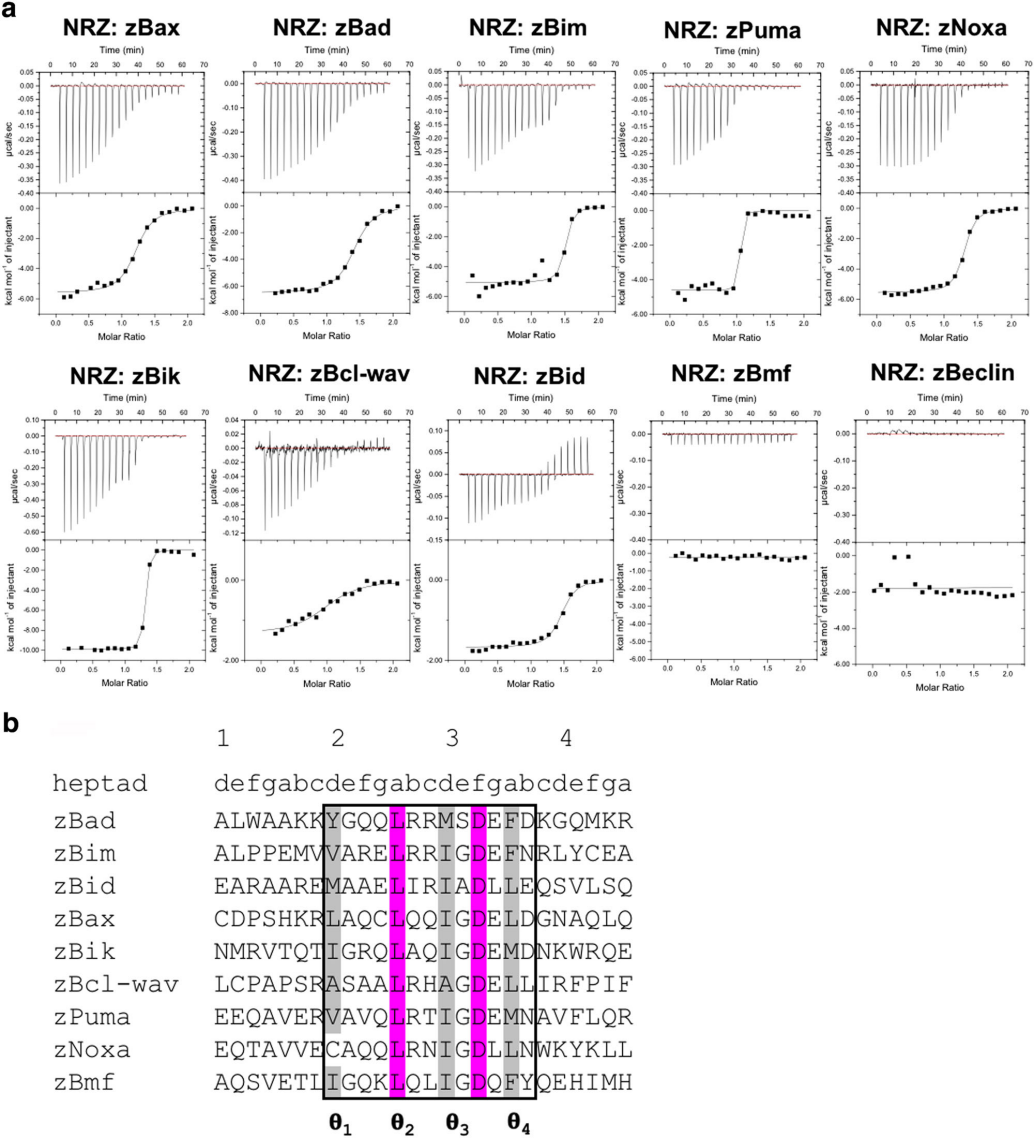
Titration curves showing the raw heats of titration for ITC measurements of NRZ: BH3 motif interactions. NRZ interacts with Bax as well as all other BH3-only proteins but not Bmf or Beclin-1. Affinities are summarized in Table 1

In order to determine the molecular basis for apoptosis control mediated by NRZ in zebrafish we systematically examined the ability of NRZ to bind to peptides spanning the BH3 motif of zebrafish encoded pro-apoptotic Bcl-2 proteins. Analysis of the *D. rerio* genome indicated that genes are present for orthologues of the mammalian pro-apoptotic Bcl-2 family members Bid, Bim, Bax, Bad, Bik, Bmf, Puma and Noxa^4,23,24,44^, as well as Bcl-wav, a pro-apoptotic paralogue unique to fish^29^. In addition a Beclin 1 orthologue, a protein that harbours a BH3-like motif that is involved in autophagy^45^ and was previously shown to interact with pro-survival Bcl-2 proteins^46^, is also present. Isothermal titration calorimetry (ITC) was used to determine the affinity of NRZ for peptides that span the BH3 motifs of zBid, zBim, zBax, zBad, zBik, zBmf, zPuma, zNoxa, zBcl-wav and zBeclin (Fig 1, Table 1). The BH3 motifs were chosen through sequence alignment with the known mammalian pro-apoptotic Bcl-2 proteins by identifying the signature sequence LXXXGDE of the BH3 motif^5^, where X is any amino acid. Interestingly, our ITC data showed that NRZ binds most BH3 motifs with the exception of those from Bmf and Beclin, which displayed no detectable affinity. Several BH3-only proteins interacted with NRZ with high affinities, including zBik (*K*_D_ 12 nM), zPuma (*K*_D_ 36 nM) and zBim (*K*_D_ 41 nM), while zNoxa (*K*_D_ 142 nM), zBad (*K*_D_ 343 nM), zBid (*K*_D_ 409 nM) and zBax (*K*_D_ 688 nM) were bound with more modest affinities. In contrast, Bcl-wav, a recently discovered novel pro-apoptotic Bcl-2 family member of zebrafish^29^ engaged NRZ with only micromolar affinity (*K*_D_ 3570 nM).

**Table 1:** ITC affinity measurements of NRZ: BH3 motif interactions

Peptide	WT NRZ *K*_D_ (nM)	NRZ R86A *K*_D_ (nM)	NRZ D79A *K*_D_ (nM)
Bax	688 ± 111	4600 ± 440	264 ± 42
Bim	41 ± 5	168 ± 8	13 ± 4
Bad	343 ± 48	4800 ± 88	3330 ± 41
Puma	36 ± 4	2770 ± 162	117 ± 5
Bik	12 ± 2	17 ± 3	14 ± 3
Noxa	142 ± 16	510 ± 41	335 ± 31
Bcl-wav	3570 ± 162	NB	NB
Bid	409 ± 55	NB	220 ± 16
Bmf	NB	NB	NB
Beclin	NB	NB	NB

26-mer peptides spanning the BH3-motyif of *D. rerio* pro-apoptotic Bcl-2 family members or Beclin-1 from zebrafish were employed. All *K*_D_ values (in nM) are the means of three replicates with standard error

*NB* no binding

**Fig. 2:**
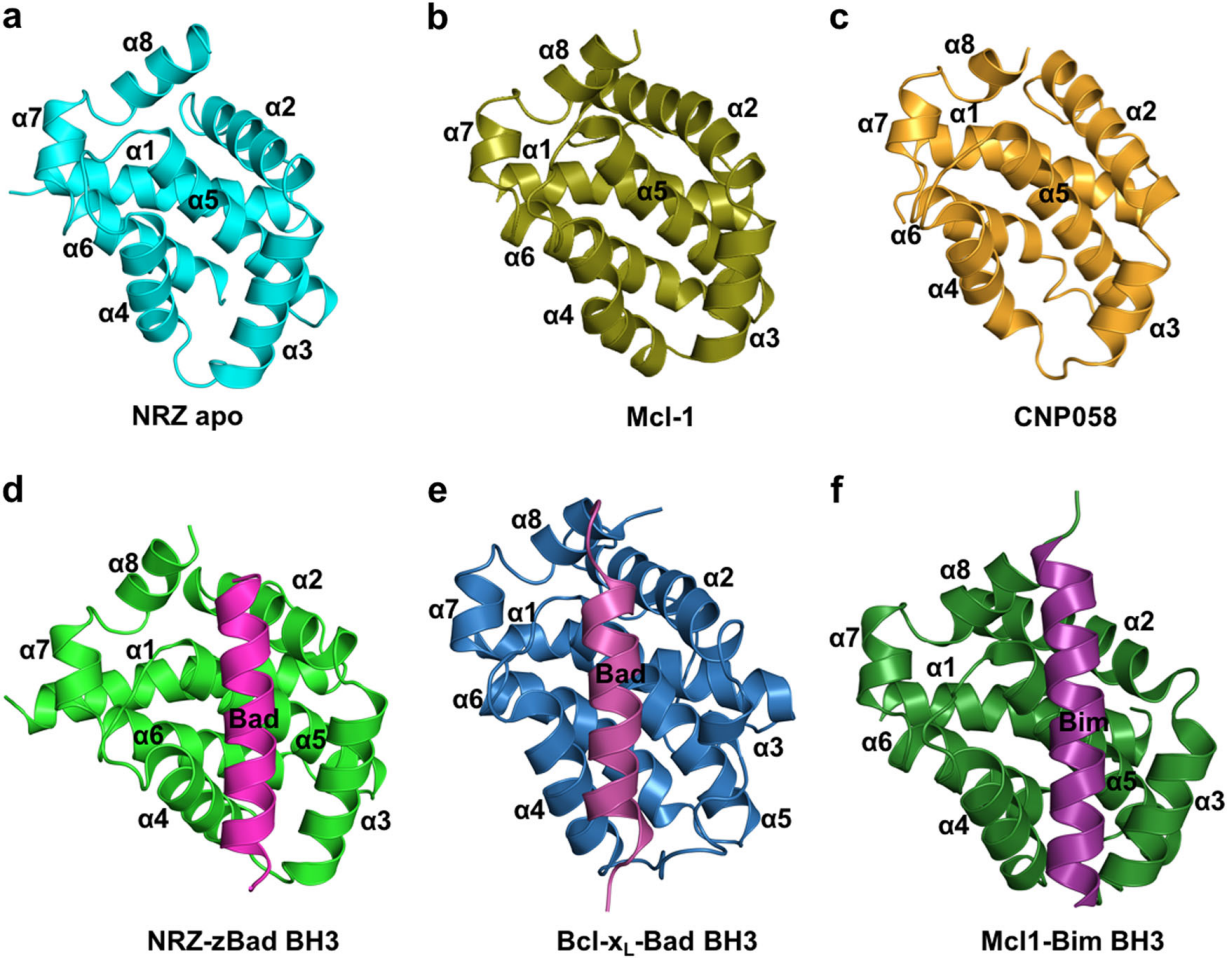
Crystal structures of NRZ, NRZ: Bad BH3 complex and comparison with closely related complexes. Cartoon representation of (**a**) *apo* NRZ (light blue). Helices are labelled as α1–8, with the view being into the hydrophobic groove formed by α2–5. **b** Cartoon representation of Mcl-1 (olive)^49^. **c** CNP058 (orange)^51^, the closest structural homolog from viral pro-survival Bcl-2 proteins for NRZ. **d** NRZ (green) in complex with zBad BH3 motif (hot pink). **e** Bcl-x_L_ (blue) in complex with Bad BH3 motif (yellow)^50^, **f** Mcl-1 (brown) in complex with Bim (magenta) BH3 motif^49^. All views in **b**–**f** are as in **a**

To determine the structural basis of NRZ interaction with BH3 motifs of pro-apoptotic Bcl-2 proteins, we determined the crystal structures of *apo*-NRZ and its complexed *holo* form bound to the BH3-motif of zBad (Fig. 2a, d, Table 2). Similar to other pro-survival Bcl-2 proteins, NRZ adopts a conserved Bcl-2-like fold consisting of eight α-helices that form a globular helical bundle. Helices α2-5 form the canonical hydrophobic ligand binding groove observed in other Bcl-2 family proteins^47^ that is utilized to accommodate the zBad BH3 peptide (Fig. 2d). An analysis using DALI^48^ showed that complexes of Mcl-1 (PDB ID 2NL9)^49^ (Fig. 2b, f) and Bcl-x_L_ (PDB ID 4QNQ)^50^ bound to Bim and Bad BH3 peptides are the closest structural Bcl-2 homologs with R.M.S.D. value 1.7 Å and 1.9 Å over 136 and 132 Cα atoms, respectively, with sequence identities of 15% and 18%, respectively. The closest viral Bcl-2 homolog to NRZ is CNP058^51^ (R.M.S.D. of 2.0 Å over 133 Cα atoms, Fig. 2c) with a sequence identity of 16%.

**Table 2:** X-ray crystallographic data collection and refinement statistics

	Native NRZ Apo	Native NRZ: Bad BH3
Data collection		
Space group	P4_3_	P6_3_
*Cell dimensions*		
a, b, c (Å)	48.18, 48.18, 75.33	87.62, 87.62, 36.77
α, β, γ (°)	90, 90, 90	90, 90, 120
Wavelength (Å)	0.9537	0.9537
Resolution (Å)	48.18–2.0 (2.07–2.0) *	43.81–1.639 (1.68–1.64) *
*R*_sym_ or *R*_merge_	0.051 (1.16)	0.092 (1.92)
*I*/*σI*	18.3 (1.7)	15.4 (1.3)
Completeness (%)	99.93 (99.57)	100 (100)
Redundancy	6.9 (6.9)	18.6 (9.9)

Refinement		
Resolution (Å)	48.18–2.0 (2.07–2.0)	43.81–1.639 (1.68–1.64)
No. of reflections	11,653	19,885
*R*_work_/*R*_free_	0.194/0.222	0.187/0.206
*No. of atoms*		
Protein	1194	1361
Ligand/ion	0	0
Water	39	105
*B-factors*		
Protein	54.98	39.6
Ligand/ion	0	0
Water	52.42	44.2
*R.m.s. deviations*		
Bond lengths (Å)	0.007	0.003
Bond angles (°)	0.84	0.51

* Values in parentheses are for the highest resolution shell.

### NRZ: BH3 motif interactions

**Fig. 3:**
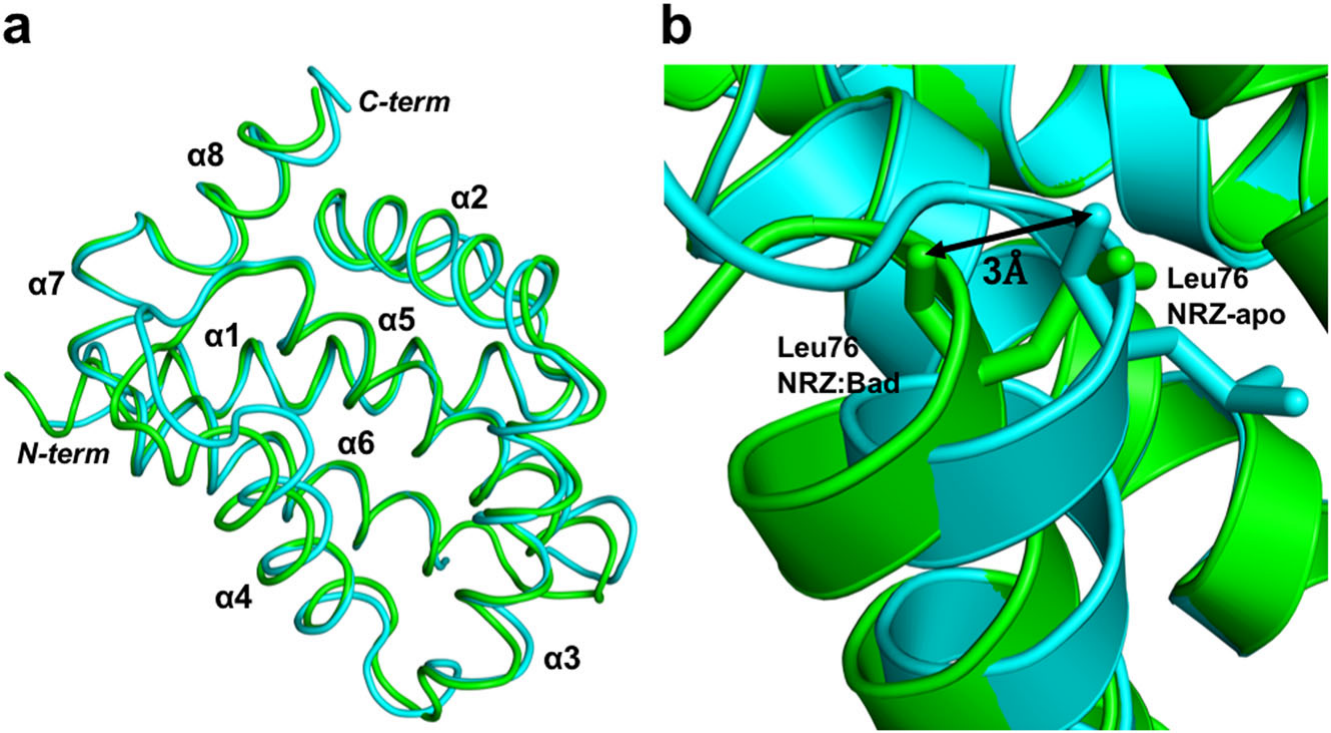
Superimposition of apo NRZ with NRZ: zBad BH3 complex. Comparison of the backbone structures of NRZ and NRZ: zBad BH3 complex. **a** Cartoon representation of *apo* NRZ (light blue) superimposed onto NRZ: zBad BH3 complex (green). The view is into the canonical hydrophobic binding groove formed by α2–5. **b** Close up view of NRZ helix α4, which is shifted by 3 Å from its original position in the apo NRZ structure upon zBad BH3 motif binding, thus enlarging the binding groove

**Fig. 4:**
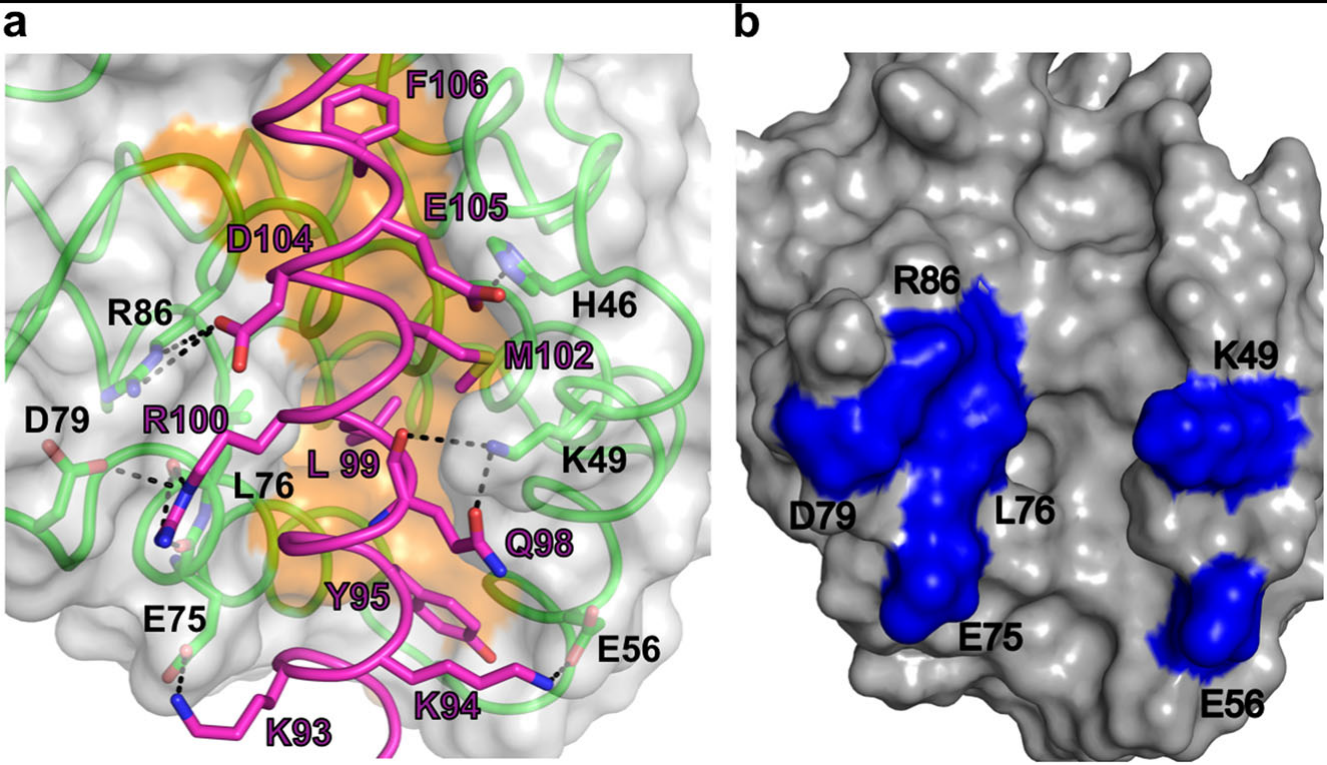
Binding of BH3 motif peptides to NRZ result in local reorganization of the canonical NRZ binding groove. ** a** Detailed view of the NRZ: zBad BH3 motif interface. The NRZ surface, backbone and floor of the binding groove are shown in grey, green and orange, respectively, while zBad BH3 is shown in hot pink. The four key hydrophobic residues of zBad (Y95, L99, M102, F106) protruding into the binding groove, and the conserved salt-bridge formed by NRZ D79 and zBad BH3 R100 are labelled, as well as all other residues involved in additional ionic interactions and hydrogen bonds. Interactions are denoted as dashed black lines. **b** Surface view of the conserved residues that are involved in NRZ-zBad BH3 interactions. Residues shown in blue are residues within canonical binding groove of NRZ (K49, E56, E75, L76, D79 and R86) that are conserved in NRZ-like proteins amongst different fish species

NRZ utilizes the canonical hydrophobic binding groove that is also found in other pro-survival Bcl-2 proteins^4^ to accommodate the zBad BH3-motif (Fig. 3a) using a combination of hydrophobic and ionic interactions as well as hydrogen bonds. To accommodate the zBad BH3-peptide NRZ undergoes localized conformational changes (Fig. 3a, d). Upon binding of the BH3-motif, the C-terminal end of α4-helix moves by 3.0 Å (Fig. 4a, b) relative to *apo*-NRZ, thus enlarging the binding groove to accommodate the Bad BH3 motif. Detailed inspection of the NRZ: zBad BH3 complex interface reveals five salt bridges between Lys93^Bad^ and Glu75^NRZ^, Lys94^Bad^ and Glu56^NRZ^, Glu105^Bad^ and His46^NRZ^, Arg100^Bad^ with Asp79^NRZ^ and Asp104^Bad^ with Arg86^NRZ^. In addition to ionic interactions, the NRZ: zBad interface also features three hydrogen bonds between Arg100^Bad^–Leu76^NRZ^, Arg100^Bad^–Glu75^NRZ^, Gln98^Bad^–Lys49^NRZ^. Finally, the four highly conserved hydrophobic residues Y95, 99L, M102 and F106 from the zBad BH3 motif protrude into the ligand binding groove of NRZ and are accommodated in four hydrophobic pockets at the floor of the binding groove.

To validate the structure of NRZ: Bad BH3 we mutated two key NRZ residues involved in ionic interactions, Asp79 and Arg86 to Ala, and examined the ability of these mutants to bind BH3-motif peptides (Table 1). Both mutants showed substantially reduced binding to Bad, with D79A displaying a tenfold reduction in Bad binding, whereas NRZ R86A displayed a 14-fold reduction in affinity. However, the contributions to binding BH3-motif peptides from these residues are not uniform across all BH3-motifs, indicating differences in the specific importance of these contacts. For example, Bik is not affected strongly by these two mutations and Bid binding is only impacted by R86A, whereas Bcl-wav binding is ablated for both R86A and D79A.

## Discussion

**Fig. 5:**
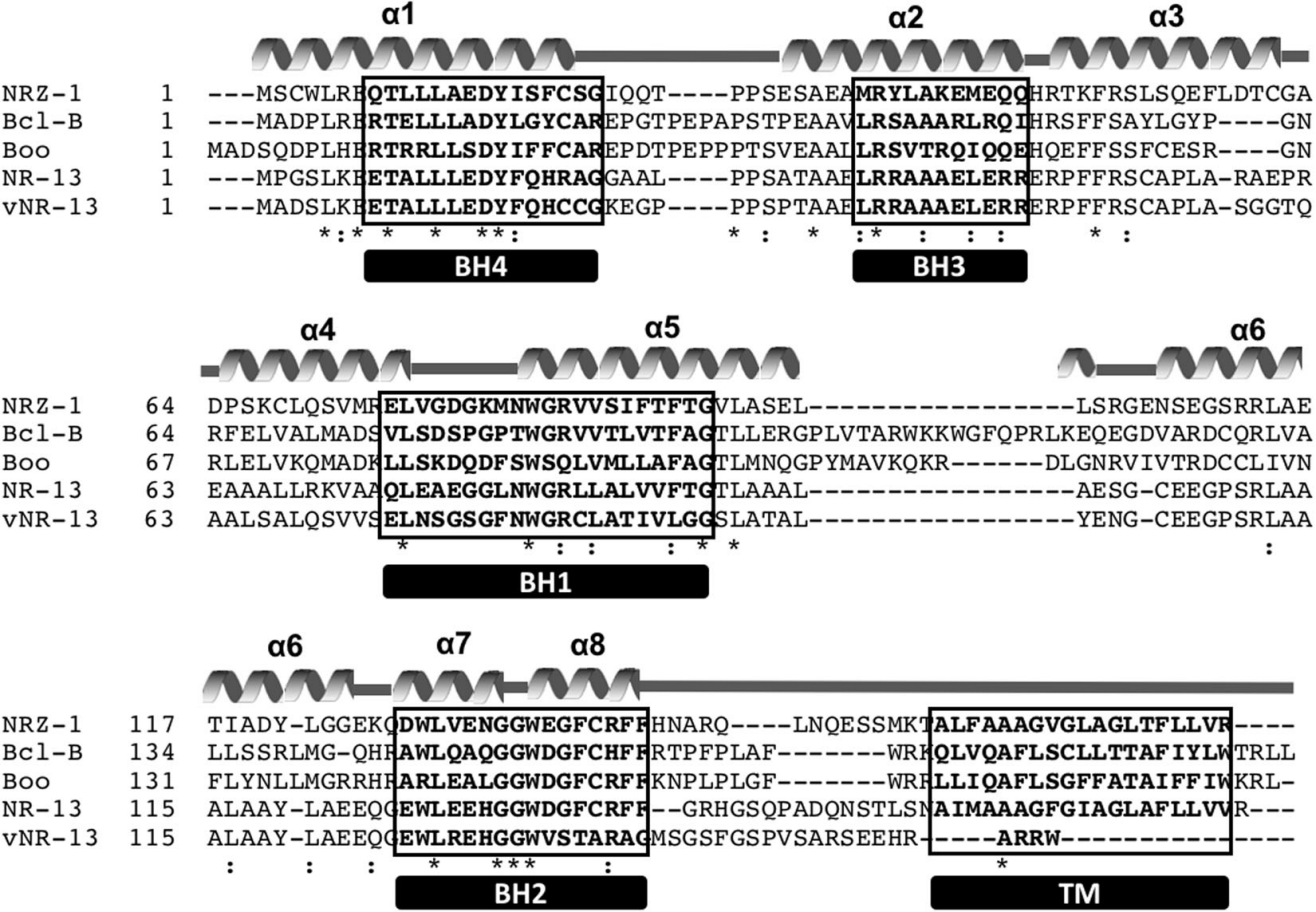
Sequence alignment of NRZ with Bcl-2 homologs from other organisms. The sequence alignment of Bcl-2 family proteins was generated with MUSCLE^66^ using sequences from zebrafish NRZ (Uniprot Accession number Q8UWD5), human Bcl-B (Q9HD36), mouse Boo (Q9Z0F3), chicken NR-13 (Q90ZN1) and herpes virus vNR-13 (Q9DH00). The α-helical secondary structure elements (α1–8) are marked as grey helices and loop regions are indicated as grey lines based on the crystal structure of NRZ. The boxed regions of the sequences are denoting the Bcl-2 homology motifs (BH motifs 1–4) and trans-membrane domains (TM) at the end of the sequences. Conserved identical residues between sequences are denoted as “*”, similar residues are denoted as “:” and semi conserved residues denoted as “.”

Developing a detailed understanding of Bcl-2 family function in apoptosis regulation is not only important for identifying their biological roles but, is crucial in the design of new therapeutic strategies directed against this family^4,52^. Indeed, as a major arbiter of programmed cell death, there is a significant interest in resolving the function of Bcl-2 family proteins at a molecular level with the aim of targeting them for their role in cancer^6,53^. Zebrafish are proving a valuable model system in this context as the mechanisms of apoptosis activation and function appear to be similar to those found in mammals^28^. Although there are many similarities between the Bcl-2 families in mammals and fish there are also significant differences that require clarification^20^. NRZ was initially identified as a *D. rerio* Bcl-2 orthologue of avian NR-13^26^ via database searches^26,28^. Sequence alignment revealed that NRZ shares 40 and 39% identity with chicken NR-13 and turkey herpes virus NR-13 with respectively. Significantly lower sequence identity is shared with the mammalian orthologues of NRZ where only 25 and 23% sequence identity are observed for the human pro-survival Bcl-2 protein Bcl-B (also known as Bcl-2 like protein 10 or NrH) and the mouse orthologue Boo (or Diva), respectively^54^. Zebrafish NRZ features significant sequence differences from other Bcl-2 proteins with only three residues identical between NRZ, Bcl-B and Boo in the region spanning helices α3–5 that constitute the canonical ligand binding groove (Fig. 5), thus potentially providing a basis for a unique ligand binding profile for this pro-survival Bcl-2 family member^4,28^. Here we examined the structure and interactions of NRZ by determining the structures of *apo*-NRZ and its complex with zBad BH3, the orthologue of mammalian Bad and measuring the binding affinities for BH3-motifs. The structures revealed the conformational changes in NRZ after binding of BH3 motif ligand and provide a structural basis for NRZ mediated apoptosis inhibition.

Surprisingly, our structural search and comparison using DALI^48^ revealed that the closest structural homolog of NRZ is in fact Mcl-1^55^ with an R.M.S.D value of 1.7 Å over 136 Cα atoms, and a sequence identity of 15%. Sequence alignment of NRZ (Fig. 5) showed that NRZ shares sequence features of other multi-domain members of Bcl-2 family. However, the BH regions show considerable sequence variation and these sequence variations that are located in the binding groove account in part for the selectivity differences observed for BH3-ligands^47^ compared to NRZ’s mammalian counterparts. Interestingly, NRZ shares only 18% sequence identity with the two zebrafish encoded Mcl-1 homologs zMcl-1a and zMcl-1b, suggesting that they may not be functionally redundant and differentially interact with pro-apoptotic Bcl-2 proteins in zebrafish.

The overall fold of the NRZ: zBad BH3 complex is very similar to that observed in other Bcl-2 complexes. Despite the overall similarity in fold, several interesting differences are observed in the crystal structures and protein:peptide interfaces of the NRZ: zBad and Mcl-1:Bim complexes. The difference in peptide binding mode of these complexes were calculated^56^ as solvent accessible surface and associated thermodynamic properties of Gibb’s free energy change (ΔG) of interface formation and dissociation. The binding of zBad to NRZ buries a total of 2366 Å^2^ solvent accessible surface and solvation energy of isolated structure −10.1 kcal/mol and ΔG of interface formation and dissociation of −3.9 kcal/mol. In contrast, binding of human Bim to human Mcl-1 buries a total of 2665 Å^2^ and solvent accessible surface and solvation energy of isolated structure −10.4 kcal/mol and ΔG of interface formation and dissociation of −4.8 kcal/mol. However, the human Bcl-x_L_:Bad complex forms a larger (total of 3268 Å^2^ and solvent accessible surface and solvation energy of isolated structure −14.4 kcal/mol and ΔG of interface formation and dissociation of −2.2 kcal/mol) ligand binding interface than that of NRZ: zBad and human Mcl-1:Bim. Structurally, NRZ features a more ordered α3 helix compared to human Bcl-x_L_, which upon Bim binding unravels the α3 helix^57^, and leads to an opening of the canonical ligand binding groove of ~9 Å due to an outward movement of α3 and a pivoting of α4. In contrast, NRZ maintains the ordered α3 helix on binding Bad, which leads to the C-terminal end of α4 helix moving by 3 Å relative to that in *apo*-NRZ (Fig. 4b).

Similar to other multi-domain Bcl-2 family proteins, including pro-apoptotic proteins Bax, Bak and Bok, NRZ also contains the highly conserved sequence motif “NWGR” as part of the BH1 motif at the N-terminal end of α5 (Fig. 2). In addition to forming a helix cap^4^ this region plays a vital role for recognition of the BH3-only proteins^58^. A hallmark of BH3 motif interactions with mammalian pro-survival Bcl-2 proteins is the formation of an ionic bond between the conserved Arginine of the “NWGR” sequence motif of pro-survival Bcl-2 proteins with the absolutely conserved Aspartate residue of the BH3 motif^58^. In the human Bcl-x_L_:Bad complex (1G5J^50^)(Fig. 3e), the corresponding R139 residue in the NWGR motif interacts with D119 of the Bad BH3 peptide^50^. Previous studies revealed that a R139Q mutation in Bcl-x_L_ results in loss of pro-survival function and ability to interact with Bax^59^. Surprisingly, this highly conserved interaction between the Arginine in the NWGR motif that is present in all other mammalian Bcl-2:BH3 complexes solved to date^4,60^ is very weak in the NRZ: zBad BH3 complex, instead an additional ionic interaction between Asp79^NRZ^-Arg100^Bad^ is observed that may compensate for the weaker and longer range canonical ionic interaction between Arg86^NRZ^-Asp104^Bad^. Mutagenesis of both Asp79 and Arg86 in NRZ indicated that indeed both contribute to the binding of Bad, with Arg86 still playing an important role in binding BH3-motif peptides despite being more distant from the partner Asp104 in Bad when compared to other pro-survival Bcl-2: BH3 peptide complexes (Table 1). Notably, the individual mutations affected binding to several BH3 motif peptides differentially, suggesting that careful mutagenesis may be utilized to probe the role of individual NRZ interactions with pro-apoptotic Bcl-2 proteins^61^.

**Fig. 6:**
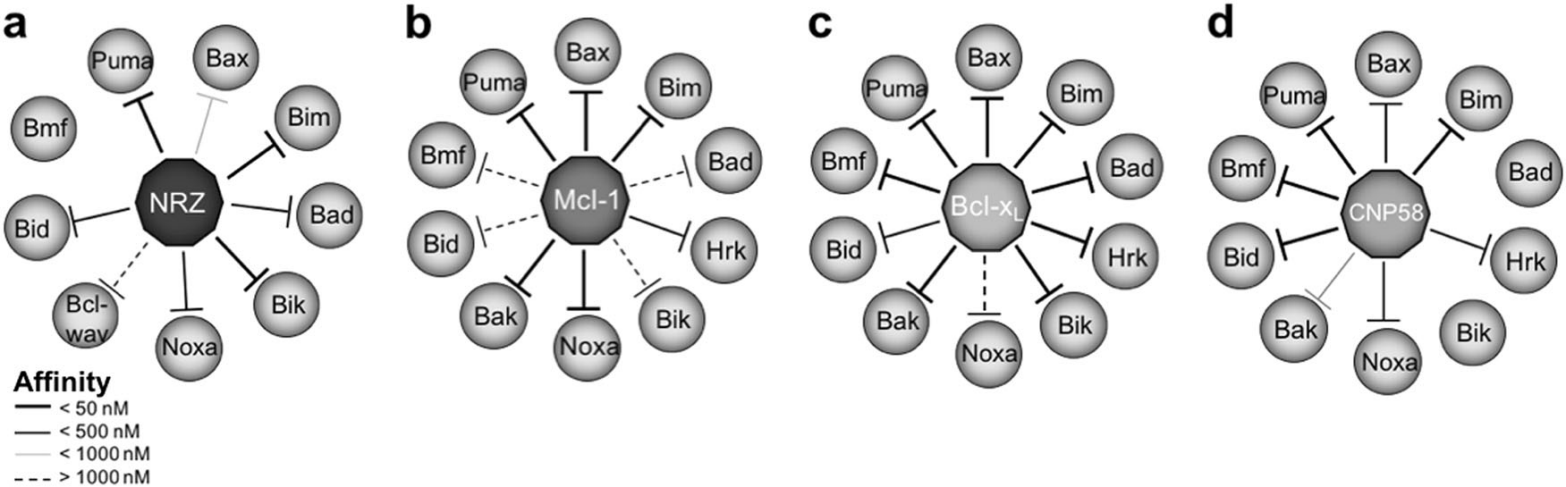
Comparison of the BH3 motif binding profile of NRZ with Mcl-1, Bcl-x_L_ and CNP058. **a** Binding profile of zebrafish NRZ with BH3 motif peptides (**b**) binding profile of human Mcl-1 (**c**) binding profile of human Bcl-x_L_ and (**d**) binding profile of canarypox virus CNP058. BH3 motif peptides used in (**b**) and (**c**) are of human origin, whereas peptides in (**d**) are from chicken. Bars indicate binding affinity ranges from <50 nM, <500 nM, <1000 nM, and >1000 nM as shown in inset

NRZ displays a very distinct ligand interaction profile when compared to its most structurally related proteins, Mcl-1, Bcl-x_L_ and CNP058. Intriguingly the sole viral Bcl-2 member encoded by a fish virus, grouper iridovirus GIV66, only binds Bim, thus displaying a radically different ligand binding profile compared to NRZ^62^. Among mammalian pro-survival Bcl-2 proteins a distinct Bad/Noxa dyad is observed, with Bcl-2, Bcl-x_L_ and Bcl-w binding Bad, but not Noxa, whereas Mcl-1 and A1 bind Noxa but not Bad^7^. In contrast, NRZ binds both Bad and Noxa with 340 nM and 140 nM affinity, respectively, a feature not previously seen outside of virus encoded pro-survival Bcl-2, with African swine fever virus encoded A179L and fowlpox virus encoded FPV039 the only known pro-survival Bcl-2 proteins that are Bad and Noxa binders^63,64^. NRZ shows no affinity for Bmf, which is bound by both human Mcl-1 and Bcl-x_L_ (Fig. 6)^7^, and overall displays a ligand binding profile that most closely resembles A1, albeit with different affinities for individual ligands^65^. Overall, the affinity measurements suggest that NRZ is unlikely to be a functional Mcl-1 homolog, as might be expected as there are two Mcl-1 orthologues in *D. rerio*, Mcl-1a and Mcl-1b^26^, and it is also unlikely to be a functional Bcl-B homolog, considering that human Bcl-B is only able to engage Bax and Bim^54^. NRZ also does not bind the BH3 motif of the autophagy regulator Beclin-1, a feature previously observed for both mammalian Bcl-2 and Bcl-x_L_^50^_,_ suggesting that NRZ does not harbour a dual role in regulating apoptosis and autophagy.

In summary, like other pro-survival Bcl-2 protein structures solved to-date, NRZ adopts a Bcl-2 like fold and its most closely related structural homologs are the cellular apoptosis inhibitor Mcl-1 and the canarypox viral Bcl-2 protein CNP058. Furthermore, we demonstrated that NRZ harbours a unique BH3 motif binding profile. However, while NRZ is a close structural homolog of Mcl-1 it seems unlikely to be a functional orthologue based on its different binding profile, in particular the ability to engage both Bad and Noxa, a feature that has not been previously observed in mammalian pro-survival Bcl-2 proteins. This study suggests that NRZ likely occupies a unique mechanistic role in zebrafish apoptosis regulation. Thus, further functional studies are required in vivo to delineate the role of NRZ in apoptosis signalling. Our findings demonstrate the complexities of delineating Bcl-2 family function and the pitfalls of assumed functional and evolutionary similarity based on sequence and structure alone.

### Data availability

The raw X-ray diffraction data were deposited at the SBGrid Data Bank (http://data.sbgrid.org)^43^ as dataset entries doi: 10.15785/SBGRID/6H1N and 10.15785/SBGRID/6FBX. The coordinates have been deposited in the Protein Data Bank (accession code 6H1N and 6FBX).
